# LncRNAs in non-small cell lung cancer: novel diagnostic and prognostic biomarkers

**DOI:** 10.3389/fmolb.2023.1297198

**Published:** 2023-12-13

**Authors:** Jiang Fu, Li Yu, Hang Yan, Shengjie Tang, Zixu Wang, Tingting Dai, Haoyu Chen, Song Zhang, Haiyang Hu, Tao Liu, Shoujun Tang, Rong He, Haining Zhou

**Affiliations:** ^1^ Department of Thoracic Surgery, Suining Central Hospital, An Affiliated Hospital of Chongqing Medical University, Suining, China; ^2^ Institute of Surgery, Graduate School, Chengdu University of Traditional Chinese Medicine, Chengdu, China; ^3^ Department of Physical Examination, Suining Central Hospital, An Affiliated Hospital of Chongqing Medical University, Suining, China; ^4^ Institute of Surgery, Graduate School, Zunyi Medical University, Zunyi, China; ^5^ Institute of Surgery, Graduate School, North Sichuan Medical College, Nanchong, China; ^6^ Department of Respiratory and Critical Care Medicine, Suining Central Hospital, An Affiliated Hospital of Chongqing Medical University, Suining, China

**Keywords:** lung cancer, lncRNA, biomarker, diagnosis, prognosis, therapy

## Abstract

Non-small cell lung cancer (NSCLC) is one of the main causes of cancer-related death worldwide, with a serious impact on human health and life. The identification of NSCLC at an early stage is a formidable task that frequently culminates in a belated diagnosis. LncRNA is a kind of noncoding RNA with limited protein-coding capacity, and its expression is out of balance in many cancers, especially NSCLC. A large number of studies have reported that lncRNA acts a vital role in regulating angiogenesis, invasion, metastasis, and the proliferation and apoptosis of tumor cells, affecting the occurrence and development of NSCLC. Abundant evidence demonstrates that lncRNAs may serve as potential biomarkers for NSCLC diagnosis and prognosis. In this review, we summarize the latest progress in characterizing the functional mechanism of lncRNAs involved in the development of NSCLC and further discuss the role of lncRNAs in NSCLC therapy and chemotherapy resistance. We also discuss the advantages, limitations, and challenges of using lncRNAs as diagnostic or prognostic biomarkers in the management of NSCLC.

## 1 Introduction

Lung cancer has the second-highest cancer incidence and accounts for the majority of cancer deaths worldwide (1,800,000 deaths per year) based on the Global Cancer Observatory in 2020 (Sung et al., 2021). According to histopathology, lung cancer is composed of two different types: small cell lung cancer (SCLC) and non-small cell lung cancer (NSCLC). Non-small cell lung cancer accounts for about 85% of the total number of lung cancers, mainly including adenocarcinoma (AC), squamous cell carcinoma (SCC) and large cell carcinoma (Wang C. et al., 2020). Most patients with NSCLC are diagnosed at a late stage since there are no specific clinical symptoms in the early stages (Tsiouda et al., 2020). Surgery, chemotherapy and targeted therapy are currently useful treatments, but the 5 years survival rate in NSCLC patients remains below 15% (Huang W. et al., 2020). Thus, patients’ survival and outcome depend on the early detection of NSCLC. Low-dose CT (LDCT) screening is the primary means of early diagnosis. However, LDCT has two disadvantages: 1) the false-positive rate is high, which can easily lead to overdiagnosis (Aberle et al., 2011), and 2) accumulated radiation from screening and follow-up causes an increased risk of cancer (McCunney and Li, 2014). The progression of NSCLC is a biological process regulated by various factors. Therefore, there is a demand to further illustrate the mechanisms of NSCLC occurrence and seek more reliable diagnostic biomarkers.

Long noncoding RNAs (lncRNAs) are a type of RNA with more than 200 nucleotides (Quinn and Chang, 2016). Due to the lack of long protein-coding open reading frames, lncRNAs are widely recognized as not having the ability to encode proteins (Zhang et al., 2023b). Emerging evidence suggests that some lncRNAs do contain small open reading frames that can be translated by ribosomes to encode peptides (Zhang et al., 2023c; Pei et al., 2023). In transcription, lncRNA is a by-product produced by RNA polymerase II, which is initially considered as a junk fragment (Entezari et al., 2022). In 1991, Borsani et al. (1991) confirmed that lncRNA XIST is correlated with the inactivation of the x chromosome. Since then, more and more studies have shown that lncRNA is essential for biological function. The abnormal expression of lncRNA usually causes various diseases, particularly malignancy (Beermann et al., 2016). LncRNA expression is differential between carcinoma and para-carcinoma tissues, suggesting its relevance to cancer occurrence and progression. For example, the expression of a novel lncRNA MCM3AP-AS1 in hepatocellular carcinoma tissues is significantly higher than that in normal liver tissues (Wang Y. et al., 2019). LncRNA-CDC6 expression increases in breast cancer tissue and its expression is closely associated with the progression of breast cancer (Kong et al., 2019).

Research on lncRNAs in recent years has shown that lncRNAs regulate target genes through epigenetics, transcriptional regulation, and post-transcriptional regulation (Zhang et al., 2021b; Du et al., 2021). Diverse biological processes can be regulated by lncRNAs, including cell proliferation, apoptosis, invasion, metastasis and drug resistance (Lin and Yang, 2018). In addition, some lncRNAs, such as HOTAIR, NEAT1, MALAT1 and MEG3, have been shown to play positive or negative regulatory roles during malignant tumor progression (Kim et al., 2018; Esposito et al., 2019). In various human body fluids, such as plasma (Lin et al., 2022), sputum (Gupta et al., 2019), saliva (Shieh et al., 2021) and urine (Huang et al., 2021), lncRNA can be detected easily and stably, and its expression varies with disease progression. Therefore, detecting lncRNA expression can be used as a new strategy for the early diagnosis and prognosis prediction of NSCLC.

In this review, we briefly describe the classification and biological functions of lncRNA and outline the roles of lncRNA in lung cancer, particularly NSCLC. We further summarize the diagnosis and limitations of lncRNAs in NSCLC. Finally, we summarize the role of lncRNAs as therapeutic targets and prognostic predictive markers in NSCLC and detail the possible challenges in this field.

## 2 Classification and molecular biological functions of lncRNA

**FIGURE 1:**
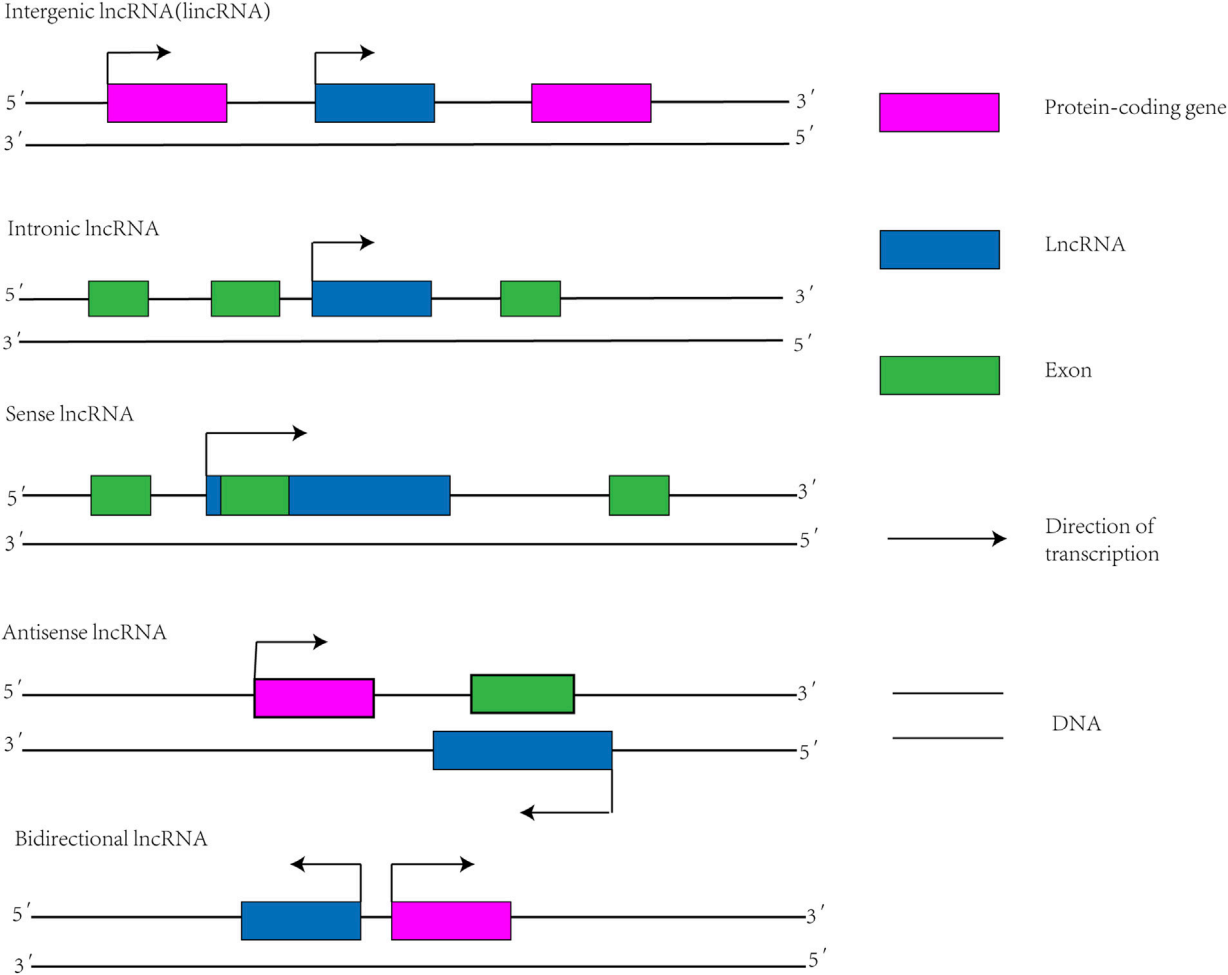
Classification diagram of lncRNAs. Intergenic lncRNA: also known as lincRNA, located between two protein-coding genes and capable of independent transcription. Intronic lncRNA: A transcript that is located in the intronic region of a protein-coding gene and has no overlap with its exon. Sense lncRNA: Transcribed from the justice chain of a protein-coding gene, overlapping with at least one exon of the protein-coding gene located on the same chain and transcribed in the same direction. Antisense lncRNA: Transcription by DNA strands complementary to protein-coding genes that are transcribed in opposite directions and overlap at least one exon of the forward gene. Bidirectional lncRNA: Shares promoters with protein-coding genes, but transcribes in the opposite direction to protein-coding genes.

Protein-coding genes make up only 2% of the human genome, while the remaining 98% do not encode proteins (Qian et al., 2019). Functional RNA that does not encode proteins is called non-coding RNA (ncRNA) (Zhang et al., 2020). Depending on the length, ncRNA is classified into small noncoding RNA and lncRNA (Li L. et al., 2021). LncRNA is transcribed by RNA polymerase II. After transcription, similar to mRNA, lncRNA is usually capped by 7-methyl guanosine (m7G) at its 5′ ends and polyadenylated at its 3′ ends (Statello et al., 2021). However, compared with mRNA, the length of lncRNA is shorter, the exons are fewer, and the primary sequence is less conservative (Lagarde et al., 2017). There are several methods used for classifying lncRNAs, one of which categorizes lncRNAs based on their location relative to protein-coding genes (Figure 1): 1) sense lncRNA; 2) antisense lncRNA; 3) intronic lncRNA; 4) bidirectional lncRNA; and 5) intergenic lncRNA (Yousefi et al., 2020).

In some studies, the function of lncRNAs is determined by their structure. Among them, secondary structures play a crucial role in lncRNAs (Derrien et al., 2012). Base complementary pairing forms the secondary structure of lncRNAs, which includes bulges, junctions, hairpin loops, stem loops, inner loops, helices, subdomains, and pseudoknots (Cruz and Westhof, 2009). Studies have shown that these secondary structures increase the stability of lncRNAs and affect their functional interactions with proteins, DNA and other RNAs (Pidíková and Herichová, 2021). For instance, MALAT1, which contains uracil-rich regions, forms a triple helix to increase stability (Zhang Y. et al., 2019). The lncRNA GAS5 signals a negative regulatory effector and has an A-type double helix structure. Its double helix structure interacts with the DNA-binding domain of the steroid receptor to repress steroid-mediated transcription (Hudson et al., 2014). These demonstrate that there is an important role for the secondary structure of lncRNAs in biological functions.

There are three main ways in which lncRNAs regulate genes: epigenetic regulation, transcriptional and post-transcriptional regulation, and their biological function depends on their location in the cell (Jia et al., 2022). In the nucleus, some lncRNAs can become histone modifiers through methylation or demethylation, thereby regulating the chromatin state and ultimately promoting or repressing gene transcription (Herman et al., 2022). LncRNA HOTAIR interacts with polycomb repressive complex 2 (PRC2) and promotes trimethylation of histone H3 Lys 27, thereby suppressing gene expression by epigenetic mechanisms (Balas et al., 2021). Moreover, the regulation of protein-coding genes by lncRNAs has also been proven through both cis-acting or trans-acting mechanisms in transcription (Ponting et al., 2009; Le Beguec et al., 2018). LncRNAs can modulate gene expression through three different regulatory mechanisms in cis-regulation: 1) lncRNA transcripts recruit specific transcription factors that regulate gene regulation. 2) LncRNAs directly regulate the expression of adjacent genes. 3) DNA sequences within a lncRNA locus can activate or repress the expression of genes in its vicinity (Merry et al., 2015). For example, XIST can silence genes present on the X chromosome by recruiting specific silencing factors (Markaki et al., 2021). In the cytoplasm, LncRNA can act as a molecular sponge for miRNA to regulate gene expression, thus reducing the targeting effect of miRNA on mRNA. This process is called endogenous competitive RNA (ceRNA) mechanism (Karreth and Pandolfi, 2013). Further, LncRNA forms a specific lncRNA protein complex (lncRNPs) with RNA binding proteins, which leads to changes in mRNA splicing and transcription and regulates signal pathways in some biological environments (Jonas et al., 2020; Cao et al., 2023). The lncRNA PTTG3P forms an RNA-protein complex with ILF3, which increases the stability of MAP2K6 and E2F1 mRNAs, thereby promoting NSCLC progression (Wang et al., 2023).

## 3 LncRNA and the occurrence and progression of NSCLC

**FIGURE 2:**
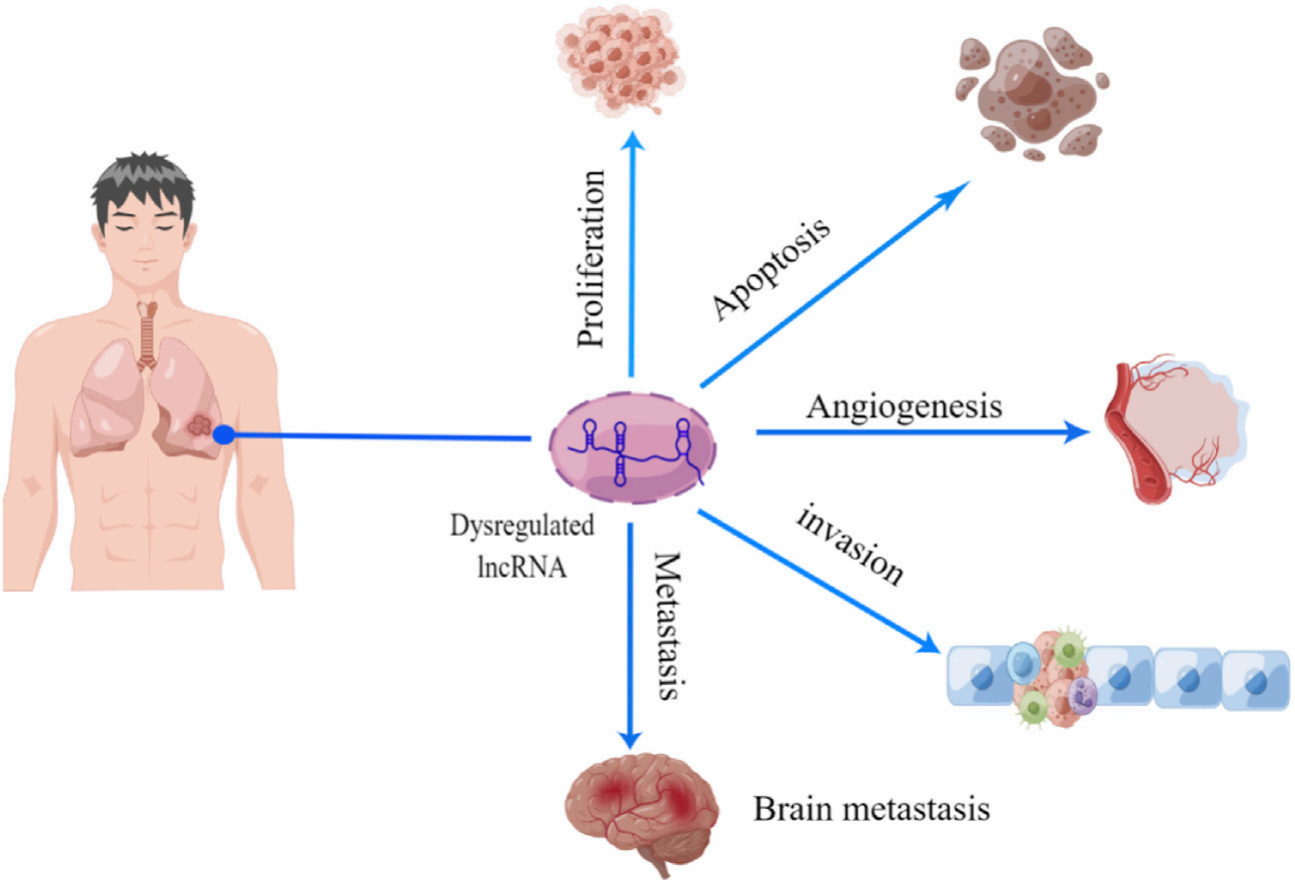
Function of lncRNA in NSCLC progression. There is growing evidence that lncRNA act as a series of novel regulators of tumorigenesis, including cell proliferation, apoptosis, angiogenesis, migration, and invasion.

The occurrence and progression of NSCLC are caused by dysregulated gene expression, usually involving oncogene activation and tumor suppressor gene inhibition. LncRNA can act as a key regulator to affect tumor cell proliferation and apoptosis, tumor angiogenesis, and tumor invasion and metastasis (Figure 2). Table 1 shows the mechanism of lncRNAs in the progression of NSCLC.

**TABLE 1:** The regulatory mechanism of lncRNA in NSCLC.

LncRNA	Expression	Model	Molecular mechanism	Function	References
HOTAIR	Up	*in vitro*	Sponges miR-149-5p to upregulate HNRNPA1	Proliferation (+)	Li et al. (2020)
GMDS-AS1	Down	*in vivo* and *in vitro*	Sponges miR-96-5p to upregulate CYLD	Apoptosis (+)	Zhao et al. (2020)
HIF1A-As2	Up	*in vivo* and *in vitro*	Promotes the expression of MYC	Proliferation (+)	Yang et al. (2023a)
BC009639	Up	*in vitro*	Regulates EMT by modulating IMPAD1	Migration (+)	Chen et al. (2023a)
H19	Up	*in vitro*	Regulates EMT	Migration (+); invasion (+)	Liao et al. (2019)
SLCO4A1-AS1	Down	*in vitro*	Sequesters the TOX4-NTSR1 signaling axis	Migration (−)	Chen et al. (2023c)
LUADT1	Up	*in vitro*	Sponges miR-15a-3p to upregulate Twist1	Migration (+); invasion (+)	Wang et al. (2019a)
Mir100hg	Up	*in vitro* and *in vivo*	Targets miR-15a-5p and miR-31-5p	Migration (+); invasion (+)	Shi et al. (2023)
LINC02159	Up	*in vitro*	Activates ALYREF/YAP1 signaling	Migration (+); invasion (+)	Yang et al. (2023b)
LETS1	Up	*in vitro* and *in vivo*	Promotes TGF-β-induced EMT	Migration (+)	Fan et al. (2023a)
TILR	Up	*in vitro*	Represses the expression of p53	Apoptosis (−)	Iwai et al. (2023)
MLETA1	Up	*in vitro* and *in vivo*	Sponges miR-186-5p and miR-497-5p	Migration (+); invasion (+)	Hsu et al. (2023)
CALML3-AS1	Up	*in vitro* and *in vivo*	Represses the expression of BTNL9	Migration (+)	Zhang et al. (2023a)
HHIP-AS1	Down	*in vitro*	Regulates HHIP mRNA	Migration (−); proliferation (−)	Hu et al. (2023)
LINC00115	Up	*in vitro* and *in vivo*	Sponges miR-154-3p to modulate Sp3	Proliferation (+); migration (+); invasion (+)	Sun et al. (2023)
AP000695.2	Up	*in vitro* and *in vivo*	Regulates the miR-335-3p/TEAD1 axis	Glycolysis (+)	Xu et al. (2023)

### 3.1 LncRNAs regulate the proliferation and apoptosis of NSCLC

The infinite proliferation of cells is one of the ten attributes of tumors (Hanahan, 2022). Several researchers verified that lncRNAs can affect NSCLC proliferation by interacting with miRNAs. For instance, an increase in lncRNA small nucleolar RNA host gene 20 (SNHG20) expression in NSCLC is associated with an unfavorable prognosis. The upregulation of SNHG20 can improve proliferation and inhibit apoptosis. Mechanistically, SNHG20 increases the expression of ZEB2 and RUNX2 through sponging miR-154 to ultimately promote NSCLC progression (Lingling et al., 2019). Metadherin (MTDH) enables cancer cells to adhere tightly to blood vessels and consequently reach other distant organs. High expression of lncRNA prostate cancer non-coding RNA 1 (PRNCR1) leads to significant overexpression of MTDH through sponging miR-126-5p to promote the proliferation of cancer cells. Conversely, the knockdown of PNCR1 promotes apoptosis and hinders proliferation in NSCLC cells. The use of miR-126-5p inhibitors eliminates this effect, suggesting that PNCR1 is able to regulate NSCLC proliferation and metastasis by sponging miR-126-5p (Guo et al., 2020). The proliferation rate of tumor cells can be boosted by the KLF12 protein. By inhibiting miR-188-5p, lncRNA DARS-AS1 can upregulate the concentration of KLF12 in cells, thereby promoting the proliferation and invasion of lung adenocarcinoma (LUAD) (Liu et al., 2021).

On the other hand, lncRNA regulates the proliferation and apoptosis of tumor cells by affecting cell cycle. Cyclin D1, as a key regulator of the cell cycle, is primarily responsible for promoting cell proliferation (Liu et al., 2018). According to some studies, the expression of lncRNA MIR503HG is downregulated in NSCLC. Using overexpression experiments, overexpression of MIR503HG inhibits cyclin D1 expression and prevents cell division in the G1 cycle, resulting in a decrease in cell proliferation (Xu S. et al., 2020). LncRNA ARAP1-AS1 is upregulated in NSCLC. A knockout of lncRNA ARAP1-AS1 can inhibit the expression of cyclin D1, thereby arresting the cell cycle at G0/G1, which significantly inhibits cell proliferation (Tao X. et al., 2020). Additionally, according to the reports of Chen et al., there is a positive relevance between the expression of the lncRNA MINCR and the proliferation of lung cancer cells, which indicates that silencing of MINCR reduces cell proliferation in PC9 cells via reducing the expression levels of cell cycle protein A, cell cycle protein D, CD4 and CDK2 (Chen et al., 2019). The overexpression of lncRNA GAN1 can induce cell apoptosis and inhibit tumor growth by arresting cells in the G0/G1 phase. In addition, GAN1 can act as a miR26a-5p sponge and upregulate the PTEN level, thus inhibiting cell proliferation and inducing apoptosis in NSCLC (Wang et al., 2021).

Abnormal proliferation of tumors is connected with abnormal energy metabolism. Under aerobic conditions, cancer cells prefer glycolysis for energy metabolism, which is less efficient in ATP and energy production compared to oxidative phosphorylation. This process of energy metabolism is called the Warburg effect (Warburg, 1956). Glycolysis is essential for cancer cell proliferation (Liberti and Locasale, 2016). The lncRNA can target miRNAs to control the glycolysis of cancer cells. The study showed that LINC00857 activates SPAG5 expression in lung tumor cells by reducing miRNA-149 expression, resulting in glycolysis and cell proliferation (Wang L. et al., 2020). In another experiment, the effect of lncRNA LINC00243 in the regulation of glycolysis is studied. Glycolysis is stimulated by the overexpression of LINC00243, enhancing lung tumor progression. By downregulating miR-507, LINC00243 positively regulates PDK4 to promote glycolysis and proliferation in NSCLC cells (Feng and Yang, 2020). In addition, lncRNA HOXA11-AS promotes PKM3 expression by binding to miR-2b-148p, thereby promoting LUAD proliferation and glycolysis (Chen W. et al., 2023).

Currently, most of the studies of lncRNA on NSCLC proliferation and apoptosis are still *in vitro* experiments. However, the mechanisms by which lncRNAs affect proliferation and apoptosis are more complex due to the tumor microenvironment, so more *in vivo* experiments are needed to validate the results of *in vitro* experiments.

### 3.2 LncRNAs regulate angiogenesis in NSCLC

When the tumor grows to a certain size, nutrition is also provided to the tumor cells through tumor vascular production to ensure the further growth of the tumor. Tumor neovascularization features high-passage, irregular vascularization, intravascular infiltration, and immature vascularization (Matsunaga and Tomita, 2020). As a result of pathological hyperplasia blood vessel abnormalities, tumor neovascularization is frequently associated with lung cancer development and occurrence. Generally, tumor angiogenesis is an intricate mechanism regulated by several angiogenic factors and signaling pathways (Nakhjavani et al., 2021), such as vascular endothelial growth factor (VEGF) (Wang et al., 2018) and the angiopoietin (Ang)/Tie2 signaling pathways. VEGF is the primary regulator that promotes the proliferation of vascular endothelial cells and can directly promote the proliferation and metastasis of tumors (Goel and Mercurio, 2013; Frezzetti et al., 2017). It has been well documented that the expression of LINC00173.v1 is upregulated in SCC and negatively connected with patient survival. Overexpression of LINC00173.v1 stimulates the proliferation of vascular endothelial cells and promotes vascular neogenesis and metastasis of SCC. Mechanistically, LINC00173.v1 promotes VEGFA expression by sponging miR-511-5p, and VEGFA acts directly on vascular endothelial cells to promote angiogenesis (Chen J. et al., 2020). During vascular remodeling, increased expression of Ang2 activates Tie2 receptors, leading to signal transduction and thereby promoting endothelial cell proliferation (Bupathi et al., 2014). Overexpression of lncRNA EPIC1 increases the density of new blood vessels in a study. Furthermore, in NSCLC, EPIC1 stimulates vascular endothelial cell proliferation via the Ang2/Tie2 axis, resulting in angiogenesis and channel formation (Hou et al., 2021).

Vasculogenic mimicry (VM) was first proposed in aggressive human melanoma by Maniotis et al. In tumor tissue, it boosts the growth, invasion, and metastasis of tumors through the rapid generation of new blood vessels (Maniotis et al., 1999; Jiang X. et al., 2020). VM was questioned by some researchers when it was first proposed in 1999, but after intensive research, the role of VM in tumor angiogenesis has been demonstrated. For example, It was found that LINC00312 is overexpressed in lung adenocarcinoma and positively affects tumor invasion and metastasis. LINC00312 can directly bind to the transcription factor Y-Box binding protein 1 (YBX1) to increase the average density of VM in lung adenocarcinoma tissue, thereby causing tumor neovascularization (Peng et al., 2018). Moreover, there is a gene-binding site for estrogen receptor beta (ERβ) on lncRNA MALAT1, and ERβ positively regulates MALAT1 through complementary pairing with the estrogen response element (ERE) on the MALAT1 promoter. Overexpressed MALAT1 targets miR-145-5p, increased the expression of neural precursor cells expressed developmentally downregulated 9 (NEDD9), and promoted VM formation and cell invasion in NSCLC (Yu et al., 2019).

To summarize, when NSCLC grows to a certain stage, it metastasizes either locally or at a distant location, and tumor angiogenesis provides suitable conditions for invasion and metastasis. Therefore, lncRNAs not only regulate tumor angiogenesis but also affect the metastasis of NSCLC.

### 3.3 LncRNAs regulate the invasion and metastasis of NSCLC

Tumor angiogenesis provides suitable conditions for tumor metastasis and invasion. Tumor invasion and metastasis caused by epithelial mesenchymal transition (EMT) can increase patient mortality (Li S. et al., 2019). EMT, first proposed by Greenberg in 1982, is a key process in cancer cell metastasis (Ashrafizadeh et al., 2020). In this procedure, epithelial cell markers are absent, such as E-cadherin. Expression of mesenchymal cell markers, such as N-cadherin, vimentin and fibronectin, is upregulated. This causes reorganization of the cytoskeleton and enhances their migratory capacity and adhesion to neighboring cells (Dongre and Weinberg, 2019). It has been demonstrated that lncRNA can influence the process of EMT. For example, according to a previous study, the levels of lncRNA XIST and ZEB2 mRNA increase in NSCLC tissues. LncRNA XIST is involved in the progression of tumors as an oncogene and may affect TGF-β-induced EMT via raising the level of ZEB2, thereby speeding up the invasion and migration of NSCLC. In addition, lncRNA XIST acts as a miRNA sponge to inhibit miR-367 and miR-141 expression. Nevertheless, overexpression of miR-141 and miR-367 block TGF-β-induced EMT, and thus the invasion and metastasis efficiency of NSCLC are reduced (Li et al., 2018). Among NSCLC tissues, Linc00460 is overexpressed, and hindering Linc00460 expression affects the expression of EMT-related proteins, thereby inhibiting cancer cell invasion and metastasis (Yue and Zhang, 2018). LncRNA CRYBG3 can directly bind to eEF1A1, promote its entry into the nucleus, and thus strengthen the transcription of MDM2. Overexpressed MDM2 binds to MDM2-binding protein (MTBP), which reduces the binding between MTBP and ACTN4, thus increasing ACTN4-mediated cell migration (Wu et al., 2021).

Abnormally expressed lncRNA takes part in the invasion and metastasis of NSCLC by regulating the expression of signaling pathway genes, such as phosphoinositide 3-kinase (PI3K) (Jiang W. et al., 2020), mitogen-activated protein kinase (MAPK) (Zhang et al., 2018), Wnt/β-catenin signaling (Liu S. et al., 2020), TGF-β/SMAD signaling (Fan et al., 2023b), and Hippo (Zeng et al., 2021). For example, there is a strong correlation between the PI3K/AKT pathway and the proliferation, differentiation, and metastasis of cells (Yu and Cui, 2016). According to some research, Fer-1-like protein 4 (FER1L4) can decrease cell proliferation and metastasis in NSCLC by hindering PI3K/Akt signaling (Gao et al., 2019). The lncRNA NEAT1 is upregulated in NSCLC tissues and cells. NEAT1 overexpression triggers invasion and migration through aiming the has-miR-376b-3p/SULF1 axis. Moreover, by participating in the phosphorylation of MAPK and Akt, NEAT1 also regulates NSCLC progression, introducing a new avenue for cancer pathogenesis (Chen L. M. et al., 2020). As a result of the sponge action of lncRNA JPX, miRNA-33a-5p expression is decreased, bringing about an increase in Twist1 expression and aiding the EMT process by activating the Wnt/β-catenin pathway. This accelerates the malignant process of NSCLC (Pan et al., 2020). Mitochondrial RNA Processing Endoribonuclease (RMRP) can recruit YBX1 to the promoter region of TGFBR1, leading to activation of the TGFBR1/SMAD2/SMAD3 pathway, which increases NSCLC cell invasion and migration (Yin et al., 2023). The lncRNA non-small cell LCAT1 (NSCLCAT1) reportedly increases the invasion and migration of cells in NSCLC by interacting with CDH1 to regulate the Hippo signaling pathway (Zhao et al., 2019).

In conclusion, invasion and metastasis are multi-step malignant processes, of which lncRNA may be one of the regulatory factors. Investigating blockers targeting lncRNA may reduce metastasis in NSCLC, thereby improving clinical treatment and patient prognosis.

## 4 LncRNAs as diagnostic markers in NSCLC

NSCLC is most commonly diagnosed at a late stage, which results in a very low survival rate for patients. The early detection and intervention of NSCLC can limit tumor advancement and improve the overall survival rate of patients. Imaging examination can be used for the early screening of NSCLC, but due to its high false-positive rate, it cannot differentiate NSCLC from benign lung lesions. Conventional tumor markers have low specificity and cannot accurately diagnose NSCLC. For example, carcinoembryonic antigen (CEA) is elevated not only in NSCLC but also in digestive tract tumors (Gao et al., 2018; Jiao et al., 2021). As a result, there is an urgent need for more specific and reliable biomarkers for the diagnosis of NSCLC. The study found that lncRNA not only exists stably in peripheral blood but also suits quantitative detection, so it may be utilized as a new molecular marker for the diagnosis of NSCLC.

Area Under Curve (AUC) is defined as the area under the ROC curve, with a value between 0 and 1. AUC provides a visual evaluation of the authenticity of the test method, and a higher AUC value indicates a higher accuracy of the test (Mandrekar, 2010). Some benign lung diseases and NSCLC have similar symptoms, such as cough and hemoptysis. Imaging features on CT are not effective in distinguishing between them, which will affect the doctor’s judgment and treatment measures. Fortunately, some lncRNAs have high value in diagnosing lung cancer and distinguishing benign lung lesions. For example, patients with NSCLC have notably upper concentrations of circulating lncRNA XLOC_009167 in their whole blood samples. Compared with healthy controls, lncRNA XLOC_009167 has an AUC value of 0.7398 for the diagnosis of lung cancer, with a sensitivity of 78.7% and a specificity of 61.8%. This suggests that lung cancer can be diagnosed by XLOC_009167. Moreover, the AUC value of XLOC_009167 in distinguishing lung cancer and pneumonia is 0.7005, the sensitivity is 90.1%, and the specificity is 50.0%. The results show that XLOC_009167 may be useful in distinguishing lung cancer from pneumonia (Jiang et al., 2018). In NSCLC patients, lncRNA ADAMTS9-AS2 is remarkably lower than in benign lung lesions or normal controls (*p* < 0.001). According to the ROC curve analysis of ADAMTS9-AS2, the AUC value of plasma ADAMTS9-AS2 for diagnosing NSCLC is 0.957, and the sensitivity (95%) and specificity (99.1%) of plasma ADAMTS9-AS2 for diagnosing NSCLC are higher than those of CYFRA 21-1 (61.3% sensitivity and 60% specificity). Additionally, ADAMTS9-AS2 expression decreases with tumor stage progression. This suggests that ADAMTS9-AS2 may be a molecular marker for early NSCLC detection (Abdul-Maksoud et al., 2021). Therefore, the discovery of lncRNA in plasma will open a new door for the early diagnosis of NSCLC.

However, some lncRNAs, when used as single diagnostic markers, are not sensitive to diagnosing NSCLC. Combining lncRNAs with conventional tumor markers is a feasible way to improve the efficiency of NSCLC diagnosis. A study found that GAS5 expression is lower in the serum of NSCLC patients (*p* < 0.001). Through ROC curve analysis, GAS5 has a higher AUC value (0.857) than CEA (0.758) in distinguishing NSCLC patients from healthy controls. Further research found that by combining GAS5 with CEA, the AUC of the combined group is 0.929, indicating that GAS5 combined with CEA can improve the positive rate of diagnosis in NSCLC patients (Li et al., 2019). In another study, the expression levels of lncRNA TBILA (*p* < 0.001) and AGAP2-AS1 (*p* < 0.001) in the serum of NSCLC patients are notably more than those of healthy controls. In addition, postoperative serum TBILA and AGAP2-AS1 levels are significantly lower compared with preoperative treatment. Through the ROC curve analysis of TBILA and AGAP2-AS1, the results show that in the diagnosis of NSCLC, TBILA has an AUC value of 0.775, AGAP2-AS1 has an AUC value of 0.734, and the AUC value of the combined serum marker Cyfra21-1 is 0.853, indicating that this combination can increase the diagnostic ability of NSCLC (Tao et al., 2020). The lncRNAs SOX2OT and ANRIL are overexpressed in NSCLC. In addition, two long noncoding RNAs and three tumor markers (CEA, CYFRA21-1, and SCCA) are used to establish an NSCLC diagnostic panel; the AUC of this combination is 0.853, the sensitivity is 77.1%, and the specificity is 79.2%. The results show that the combination has a significantly greater power of diagnosis for NSCLC than lncRNA alone (Xie et al., 2018).

There is evidence that combinations of lncRNAs are more accurate than individual lncRNAs in diagnosing NSCLC. For example, the AUC value of the combined application of GAS5 and SOX2OT is 0.902, and the sensitivity and specificity reach 83.8% and 81.4%, which are more than those of GAS5 and SOX2OT alone (Kamel et al., 2019). NSCLC patients express more SNHG1 and RMRP in plasma than the control group (both *p* ≤ 0.05), and among the development cohort, compared with either gene alone, these two genes together have a diagnostic sensitivity of 84.13% for NSCLC, thereby improving the rate of diagnosis of NSCLC (Lin et al., 2018). Four lncRNAs (RMRP, NEAT1, TUG1, and MALAT1) are screened using qRT-PCR in 265 plasma samples (including 148 NSCLC and 117 controls) with differential expression levels between NSCLC and controls. A combination of four lncRNAs is established, and an ROC analysis is conducted to evaluate the diagnostic performance of the established four-lncRNA panel. The AUC of the combination for diagnosing NSCLC is 0.86, and this AUC value is significantly higher than the AUC value of an individual lncRNA. Further research found that the four-lncRNA panel also provides the ability to differentiate between certain benign diseases, including COPD, tuberculosis, and inflammation of the lung (Yuan et al., 2020).

As mentioned earlier, lncRNAs (Table 2) have high diagnostic efficiency and are promising markers for the diagnosis of NSCLC. However, these studies also have certain limitations. First, the number of samples used is not sufficient. In future experiments, it will be necessary to expand the sample size, conduct further multi-center cohort studies, and use an independent database for external validation. Second, the mechanism of the dysregulated expression of lncRNAs is still unclear, and exploring this mechanism will help to establish new diagnostic markers. Finally, lncRNA expression levels must be examined in other cancers to screen for the most specific lncRNAs in NSCLC diagnosis. The new model of combining lncRNA with chest CT and traditional tumor markers may be further used in the future for the diagnosis of NSCLC.

**TABLE 2:** LncRNAs as diagnostic markers in NSCLC.

LncRNA	Expression	Sample	Method	AUC	Sensitivity (%)	Specificity (%)	References
DLX6-AS1	Up	Serum	qRT-PCR	0.806	77.5	85.9	Zhang et al. (2019a)
MAGI2AS3 and ZFAS1	Down	Plasma	qRT-PCR	0.902	N/A	N/A	Luo et al. (2018)
RP5-977B1	Up	Serum	qRT-PCR	0.8899	82.86	84.93	Min et al. (2022)
LINC-PINT	Down	Serum	qRT-PCR	0.873	90.9	75.8	Zhang et al. (2021a)
HEIH	Up	Peripheral blood	qRT-PCR	0.860	72.86	95.71	He et al. (2022)
LINC00313	Up	Serum	qRT-PCR	0.916	78.91	90.63	Wang et al. (2022b)
LINC00173	Up	Serum	qRT-PCR	0.809	62.96	89.01	Yang et al. (2020)
RP11-438N5.3	Down	Plasma	qRT-PCR	0.814	N/A	N/A	Chen et al. (2020c)

## 5 LncRNAs as prognosis markers in NSCLC

The prognosis of patients is closely related to TNM staging and the treatment of NSCLC. The overall survival rate of NSCLC patients is low because of drug resistance. At present, there is no accurate method to evaluate the prognosis of NSCLC. It has been found that lncRNAs can predict lymph node metastasis and TNM staging. Therefore, lncRNAs can be used as potential prognostic markers for NSCLC. Researchers revealed that lncRNA AC099850.3 is greatly upregulated in LUAD. Through Cox multivariate regression analysis, the results show that lncRNA AC099850.3 is an independent prognostic indicator that is associated with overall survival (OS), disease-free survival (DSS), and progress-free survival (PFS) among patients with LUAD (Chen et al., 2022b). LncRNA DPP10-AS1 expression is higher in 94 lung cancer tissues compared with normal tissues. DPP10-AS1 promotes the proliferation of lung cancer cells, which leads to a poor prognosis in patients. It is possible to use DPP 10-AS1 as an independent prognostic predictor and to determine a patient’s prognosis (Tian et al., 2021). Recently, lncRNA has been increasingly proven to have the potential for tumor prognosis. The level of lncRNA KTN1-AS1 is correlated with TNM stage (*p* = 0.0029), histological grade (*p* = 0.012) and lymph node metastasis (*p* = 0.020), and the high expression of KTN1-AS1 can reduce the OS of NSCLC patients (Liu et al., 2020). There is a shorter overall survival for NSCLC patients with high expression of the lncRNA PTTG3P. Moreover, the total survival of the high-expression group of PTTG3P in females and males with NSCLC is shorter than the low-expression group, and there is also a correlation between PTTG3P and DFS (Huang et al., 2020).

These results reveal that lncRNAs can serve as a prognostic predictor of tumors, and their expression level in tumors can be used to evaluate the clinicopathological features and overall survival of patients. Table 3 summarizes lncRNAs that affect the prognosis of NSCLC.

**TABLE 3:** LncRNAs as prognostic markers in NSCLC.

LncRNA	Expression	The HR of lncRNA expression		Prognosis (if lncRNA is upregulated)	Function	References
		Univariate analysis	Multivariate analysis			
SLC16A1-AS1	Down	3.858	3.351	Good	Proliferation (−)	Liu et al. (2020b)
RFPL3S	Up	N/A	N/A	Poor	Malignant progression (+)	Liu et al. (2020d)
LINC00504	Up	3.261	2.895	Poor	Malignant progression (+)	Ma et al. (2020)
ZEB2-AS1	Up	N/A	N/A	Poor	Migration (+); invasion (+)	Xu et al. (2020a)
LINC00847	Up	N/A	2.896	Poor	Migration (+)	Li et al. (2021a)
CCDC144NL-AS1	Up	N/A	2.577	Poor	Proliferation (+); migration (+)	Zhang et al. (2021c)
AL139385 1	Up	1.317	1.254	Poor	Proliferation (+); migration (+)	Chen et al. (2022a)
AC020978	Up	2.066	1.763	Poor	Migration (+); invasion (+)	Xu et al. (2021)
LINC00342	Up	N/A	N/A	Poor	Proliferation (+)	Tang et al. (2019)
KCNQ1OT1	Up	N/A	N/A	Good	Proliferation (−)	Sun et al. (2018)
LCAT1	Up	N/A	N/A	Poor	Proliferation (+); migration (+); invasion (+)	Yang et al. (2019)
SNHG16	Up	N/A	0.154	Poor	Migration (+); invasion (+)	Han et al. (2019)
MNX1-AS1	Up	N/A	N/A	Poor	Migration (+)	Liu et al. (2019)
CASC15	Up	2.039	1.901	Poor	Migration (+)	Li et al. (2019b)
EGFR-AS1	Up	N/A	1.218	Poor	Proliferation (+)	Xu et al. (2019)
TBULC	Up	N/A	N/A	Poor	Migration (+); invasion (+)	Zheng et al. (2019)
FAM138B	Down	N/A	3.077	Good	Proliferation (−); migration (−)	Gao et al. (2023)

## 6 LncRNA and the treatment of NSCLC

Despite the rapid progression of chemotherapy and targeted therapies in the treatment of NSCLC, the appearance of drug resistance is inevitable. Emerging evidence suggests that dysregulation of lncRNAs plays a crucial role in the development of drug resistance in NSCLC cells. Therefore, targeting dysregulated lncRNAs may provide a novel therapeutic strategy for NSCLC.

### 6.1 LncRNAs regulate drug resistance of NSCLC

Cisplatin (DDP) is one of the basic chemotherapy drugs for the treatment of NSCLC due to its broad and strong anti-cancer effect (Zhou et al., 2018; Arbour and Riely, 2019). However, cisplatin-based chemotherapy still faces significant challenges owing to acquired drug resistance (Huang et al., 2019). Hence, it is vital to learn the mechanisms of cisplatin resistance to improve the efficacy of clinical treatment. Several studies have revealed that lncRNAs are key regulators of resistance to chemotherapy drugs. For example, lncRNA LINC01116 has a high expression in patients with lung adenocarcinoma, and the dysregulated expression of lncRNA LINC01116 leads to the resistance of lung adenocarcinoma to cisplatin through the EMT process. Conversely, LINC01116 knockdown may enhance sensitivity to cisplatin by regulating apoptosis and blocking the cell cycle (Wang et al., 2020). As lncRNA ZXF1 activates the MAPK signaling cascades of ERK, JNK and p38, it promotes cisplatin resistance and cancer progression, leading to treatment failure and tumor recurrence (Yu et al., 2020). According to another study, LINC01224 may compete with miR-2467, promoting tumor progression and increasing DDP resistance in NSCLC (Xiao et al., 2021).

In recent years, molecular targeted therapy has emerged as a significant approach to treat NSCIC. However, the emergence of resistance to targeted therapies in NSCLC is inevitable. lncRNAs can affect the formation of drug resistance mechanisms for targeted therapy. Increasing lncRNA UCA1 expression can make NSCLC cells more resistant to gefitinib, and the mechanism of occurrence is that lncRNA UCA1 upregulates the expression of FOSL2 by acting as a molecular sponge of miR-143, thus resulting in enhanced resistance to gefitinib in NSCLC cells (Chen et al., 2020). In addition, the lncRNA CCAT1 can reduce the expression level of miR-218, further upregulating HOXA1 expression and correspondingly promoting gefitinib resistance in NSCLC (Jin et al., 2020). Drug resistance in NSCLC is correlated with EZH2, which is the core subunit of polycomb repressive complex 2 (PRC2) (Zhan et al., 2019). By binding to EZH2, the lncRNA CASC9 inhibits the expression of DUSP1 in gefitinib-resistant PC9/GR cells, thus increasing the drug resistance to gefitinib (Chen et al., 2020). LncRNA LINC00969 inhibits the activation of the NLRP3/caspase-1/GSDMD-associated cellular pyroptosis signaling pathway, thereby promoting gefitinib resistance in NSCLC cells (Dai et al., 2023).

Overall, lncRNAs play a key role in drug resistance in NSCLC, but the mechanism of drug resistance is more complex. Knockdown or enhancement of lncRNA expression may promote drug sensitivity in cancer cells, thus lncRNAs are promising therapeutic targets.

### 6.2 LncRNAs as therapeutic target in NSCLC

A number of lncRNAs have increased or decreased expression in NSCLC tissues. Therefore, inhibition of oncogenic lncRNAs or promotion of anticancer lncRNAs can be one of the means of treatment for NSCLC. Overexpression of the lncRNA SNHG18 can stimulate NSCLC proliferation and invasion. In a mouse model, when SNHG18-knockdown NSCLC cells are transplanted into nude mice, tumor weight and metastatic potential are lower than in the control group (Fan et al., 2021). LncRNA HOXC-AS3 is connected to the occurrence of various cancers; there is evidence that HOXC-AS3 expression is up in NSCLC tissues and cells. Knockdown of HOXC-AS3 inhibits tumor progression and reduces the proliferation rate of cancer cells, and *in vivo* in the nude mice xenograft model, HOXC-AS3 knockdown consistently reduces tumor volume and weight compared to controls (Su et al., 2022). In addition, Wang et al. reported that overexpression of lncRNA ZNRD1-AS1 diminishes the proliferation of H1299 cells, and ZNRD1-AS1 overexpressing mice has lower tumor weights compared to the blank group, which is the same as the results of *in vitro* cellular studies (Wang et al., 2022a).

Tumor drug resistance is one of the difficulties in the therapeutic process. Knockdown or overexpression of lncRNAs can recover the sensitivity of cancer cells to drugs, thus improving drug efficacy. For example, linc00665 expression is upregulated in NSCLC tissues. When linc00665 is knocked down, the number of H1975 and H1299 cells is greatly reduced by the same concentration of DDP. In a mouse model, the knockdown of linc00665 also increases the sensitivity of H1299 cells to DDP (Yang et al., 2021). Furthermore, the lncRNA APCDD1L-AS1 expression in Icotinib-resistant cells is also significantly elevated. Knocking down the expression of APCDD1L-AS1 in Icotinib-resistant cells induces a marked reduction in the protein and phosphorylation levels of EGFR and significantly increases the reaction of lung adenocarcinoma cells to Icotinib (Xie et al., 2021). Thus, lncRNAs can be emerging therapeutic targets for NSCLC.

## 7 Conclusion

There is a high mortality rate associated with NSCLC, which is a highly malignant tumor. The survival rate of malignant tumor patients can be improved by early screening and diagnosis. However, there are no effective screening methods for NSCLC at an early stage. Researchers detected dysregulation of lncRNA expression in NSCLC tissues. This article reviews the role of lncRNA in regulating the progression of NSCLC and its influence on the diagnosis, treatment, and prognosis of NSCLC. First, lncRNAs regulate cells’ proliferation and apoptosis in three different ways: 1) lncRNAs can act as ceRNAs and then regulate the expression of related proteins; 2) lncRNAs cause cell division arrest in the G1 phase, thereby affecting cell proliferation and apoptosis; and 3) lncRNAs regulate the glycolytic pathway. As the tumor grows, lncRNA expression abnormally contributes to the proliferation of vascular endothelial cells by affecting the secretion of angiogenic factors by tumor cells and regulating the signaling of Ang/Tie2, which affects the growth and progress of NSCLC. In addition, tumor angiogenesis is also influenced by VM. Secondly, the metastasis and invasion of lung tumor cells are regulated by lncRNAs, which can induce EMT and enhance the metastasis and invasion of NSCLC. lncRNAs can also promote or inhibit EMT by regulating the transduction of various signal pathways. Finally, compared with the paracancerous tissue, there is a significant upregulation or downregulation of many lncRNAs found in tumor tissues, and their expression differences make lncRNAs potential diagnostic markers of NSCLC. Although some lncRNAs are effective at diagnosing NSCLC, clinical diagnosis generally relies on a joint approach. Combining lncRNAs with conventional tumor markers can improve diagnostic performance. LncRNAs can make lung tumor cells resistant to cisplatin, gefitinib, etc. Restoration of dysregulated lncRNA expression improves cancer cell responses to chemotherapy. Gene therapy targeting lncRNAs is a new strategy. Some lncRNAs have a correlation with lymph node metastasis and TNM stage, suggesting that lncRNAs can also be used as prognostic indicators. In summary, lncRNA is linked to the onset and development of NSCLC and may be taken as an indicator of diagnosis and prognosis.

## 8 Challenges and prospects

LncRNAs can exist stably in human body fluids and show specific expression profiles in various types of NSCLC, which provides a potential choice for the diagnosis of NSCLC. Some lncRNAs are expressed at opposite levels in tissue and serum, which may be related to the sample type (tissues and serum), the demographic feature of the study population (race, region, etc.), and the detection methods used. It was found that the same lncRNA is expressed to opposite degrees in different cancers, which indicates that the potential molecular mechanism of lncRNAs in cancer progression is more complicated. There are always differences between the results of some *in vitro* experiments and clinical phenomena because *in vitro* experiments cannot completely simulate anti-tumor immunity in the human body. Therefore, higher-quality studies are needed in the future to explore the mechanisms of lncRNAs in NSCLC development, create combinations of lncRNAs with higher specificity and conduct comprehensive validation in large-scale studies. In the future, some lncRNA-related clinical trials will be conducted to validate the results of *in vitro* experiments and animal models.
